# Applying Bloom’s Taxonomy to Student Outcomes in a Flipped Classroom Model

**DOI:** 10.1007/s40670-026-02840-2

**Published:** 2026-07-30

**Authors:** Manav Shah, Dasha Musatova, Jeffrey B. Bird, Doreen M. Olvet, Joanne M. Willey

**Affiliations:** 1 Northwell, New Hyde Park, NY USA; 2 Donald and Barbara Zucker School of Medicine at Hofstra/Northwell, 500 Hofstra University, Hempstead, NY 11709 USA

**Keywords:** Flipped classroom, Bloom’s taxonomy

## Abstract

This study compared medical student learning across Bloom’s cognitive levels when content was taught using a traditional lecture or a flipped classroom model. Open-ended examination questions classified as foundational, application, or problem-solving were compared between second-year medical students who experienced a flipped classroom model in a microbiology course (2022 and 2023, *N* = 204) to those in a traditional lecture format (2020 and 2021, *N* = 200). There was no significant difference in scores for foundational questions, but significantly higher scores on application and problem-solving questions, suggesting the flipped classroom enhanced students’ depth of learning. Most students (88%) viewed the flipped classrom approach positively.

## Introduction

The flipped classroom model has gained popularity in undergraduate medical education (UME). In this approach, students engage with learning material prior to attending class and use in-class time for student-centered learning activities with the goal of promoting application and critical thinking [1]. Success relies on prework consisting of course director-produced videos or other curated materials that presents content traditionally delivered during lecture. Ideally, prework is linked to classroom activities requiring higher-order cognitive processes [2]. This can be conceptualized by Bloom’s taxonomy [3], with prework focusing on foundational skills such as remembering, while class time is spent applying and analyzing, or even evaluating and creating.

Current literature indicates that the flipped classroom often outperforms a traditional lecture approach across multiple metrics, including greater student engagement [4–6] and improved student learning [1, 5, 7, 8]. Improvements on student examination outcomes across disciplines including physiology, pharmacology, and neurophysiology are also reported [1, 7, 8]. However, not all studies have found such gains. Some have reported that it does not lead to higher test performance, suggesting that outcomes may depend on contextual factors such as design and assessment type [9]. A significant barrier to flipped classroom success is that students do not always complete the required prework, undermining in-class activities [9, 10]. Additionally, assessments in UME frequently rely on multiple-choice examinations, which may emphasize lower cognitive domains of Bloom’s taxonomy [11]. Literature evaluating the ability of the flipped classroom to improve student learning with respect to higher cognitive domains [3, 12–14] is limited. We hypothesized that students who learn content in a flipped classroom setting would outperform students who learned the same content through traditional didactic teaching in this domain.

## Methods

This study was conducted during an 8-week microbiology course at the Donald and Barbara Zucker School of Medicine at Hofstra/Northwell (ZSOM). We compared second-year medical students who participated in the course through traditional lecture in 2020 (*N* = 98) and 2021 (*N* = 102) with those who participated in flipped classroom sessions in 2022 (*N* = 101) and 2023 (*N* = 101). All classes throughout the study period were in person; attendance ranged from 70 to 85%.

Historically, this course was taught using a traditional didactic model, employing Socratic questioning [15] as its primary means of student engagement. Assigned prework included textbook readings and short, third-party videos. In 2022, the flipped classroom approach was implemented to teach the same content using course director-produced prework videos coupled with in-class sessions involving application and problem-solving exercises. Videos presented foundational, first-order information that students were expected to review prior to class. Every video was linked to 10 optional multiple-choice questions designed to reinforce the material. Each in-class session had between 1 and 3 corresponding videos of 10–20 min in length. At the start of in-class sessions, student preparation was appraised using an audience response platform, and gaps in understanding were addressed. At the end of each session, instructors highlighted key takeaway facts and concepts and answered questions.

During the last week of the course, a summative 4-hour exam consisting of approximately 40 open-ended questions was administered to all students. Course directors mapped each exam item to one of three Bloom’s taxonomy categories: remembering (foundational questions; 10–15% of questions), apply/analyze (application questions; 70–80% of questions) and evaluate/create (problem-solving questions; 5–10% of questions) by analyzing the task students were asked to perform. For example, questions that stated “list” or “state” were considered foundational; application questions asked students to analyze data or “compare and contrast”; problem-solving questions required integration and analysis of more than one concept to reach a specific conclusion. Despite differences in some questions annually, identical learning objectives were assessed throughout the study period. The final exam was graded by the same team of faculty experts each year. Graders used a scoring rubric to assign full, partial, or zero credit [16]. All exams and responses were de-identified.

Following completion of the course, all students completed a required, anonymous quantitative and qualitative end-of-course evaluation. One survey question asked students to rate their agreement with the following statement: “I found the suggested resources/prework useful for large group sessions.” Data from this question was available for students in 2021 (traditional) and 2022 (FC). Starting in 2022, surveys also queried students’ perception and satisfaction with the flipped classrom. Surveys used a 5-point Likert scale for all questions (1 = strongly disagree, 2 = disagree, 3 = neutral, 4 = agree, 5 = strongly agree).

### Statistical Analysis

Data were statistically evaluated using IBM SPSS Statistics (SPSS Inc., Chicago, Illinois, USA, Version 28.0). Descriptive statistics are presented as the mean (and standard deviation) for continuous data and number (percent) for ordinal data. Student scores were calculated as percent correct for each question type (foundational, application, and problem-solving) and an independent sample t-test was conducted to compare traditional vs. FC cohorts. The Mann-Whitney U test was used to compare differences between groups on the survey question. Effect sizes were reported for the t-test (Cohen’s d [95% confidence interval]) and Mann-Whitney U (rank-biserial correlation, r). A *p* value ≤ 0.05 was considered statistically significant. This study was deemed exempt from review by the Hofstra University Institutional Review Board (HUIRB Approval Ref#: 20240502-SOM-WIL-1). No student names, personal information, or other identifiers were available to the researchers.

## Results

**Fig. 1:**
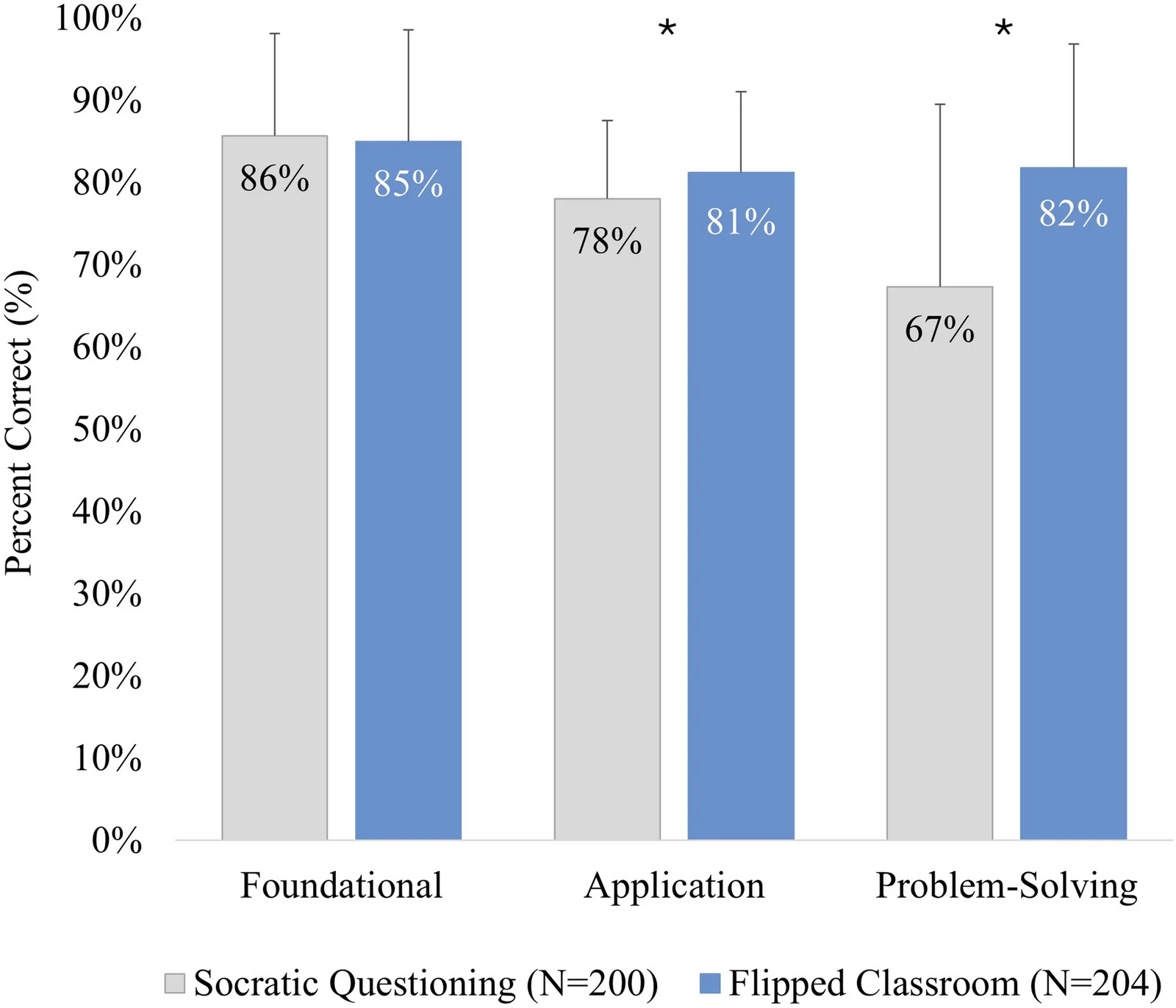
Mean percent (and standard deviation) scores of students in the traditional Socratic questioning (gray; *N* = 200) and flipped classroom curriculum (blue; *N* = 202) for the foundational, application, and problem-solving questions. The flipped classroom group performed better than Socratic questioning group on application (*p* = 0.001) and problem-solving questions (*p* < 0.001), but there was no significant difference in performance on the foundational questions (*p* = 0.61). *p ≤ 0.001

A total of 202 students participated in flipped classroom sessions, and 200 students participated in traditional sessions. As shown in Fig. 1, among foundational question responses, there was no difference in performance between the two groups (85% flipped vs. 86% traditional; t(400) = 0.51, *p* = 0.61; Cohen’s *d* = 0.05 [0.03–0.07]). Students in the flipped classroom group scored significantly higher than those in the traditional group on the application (81% vs. 78%; t(400)=-3.30, *p* = 0.001; Cohen’s *d* = 0.33 [0.32–0.34]) and problem-solving questions (82% vs. 67%; t(400)=-7.66, *p* < 0.001; Cohen’s *d* = 0.77 [0.75–0.80]).

**Fig. 2:**
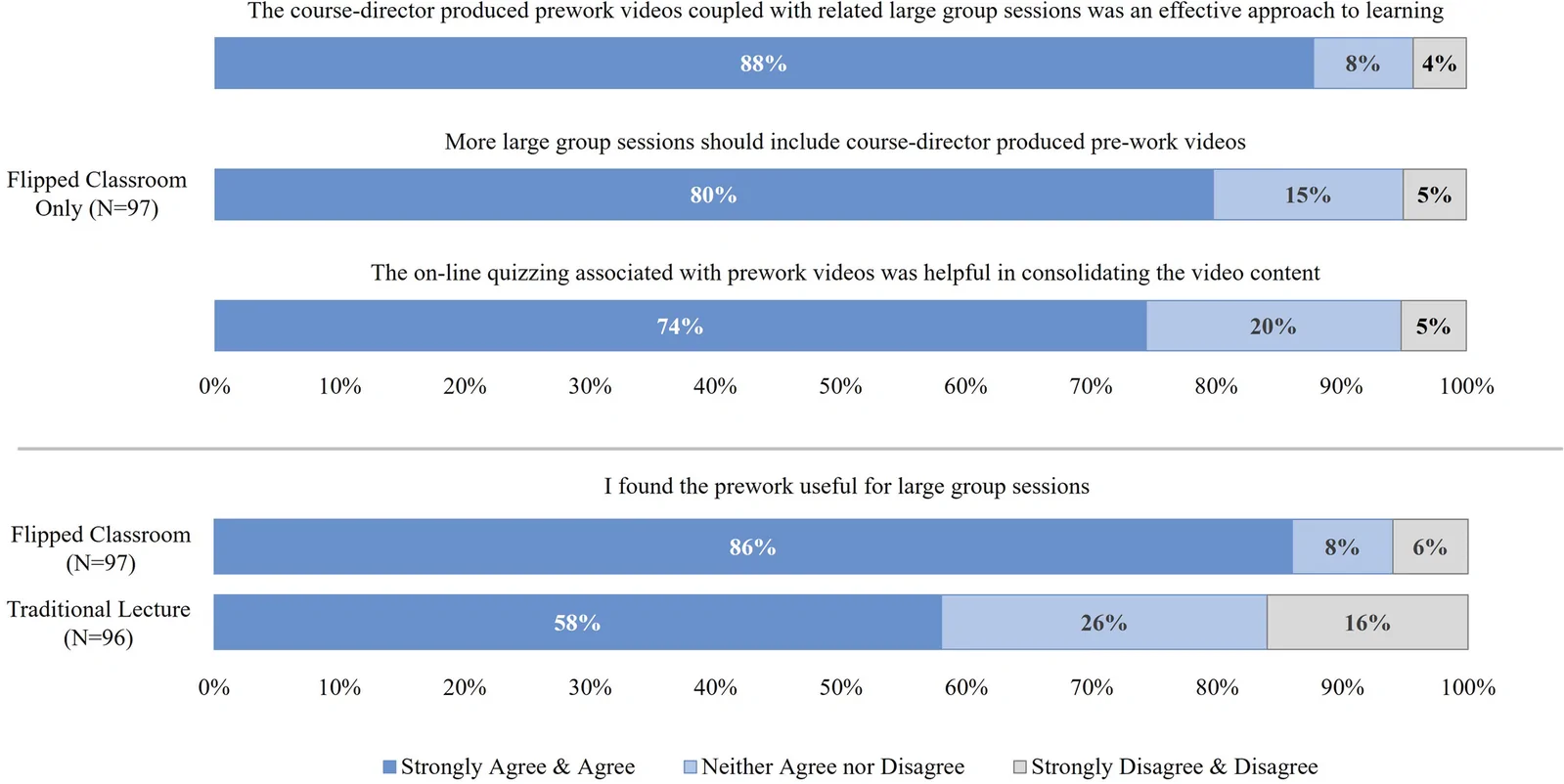
Student perception data presented as the percent of students who agreed (strongly agreed and agreed), disagreed (strongly disagreed and disagreed) or responded neither agreed nor disagreed to each statement. Top: Data was available from the 2022 and 2023 cohorts (flipped classroom only, *N* = 188). Bottom: Data was available from the 2021 (*N* = 96, traditional lecture) and 2022 (*N* = 97, flipped classroom) cohorts

Post-course survey data was available from the 2021 (*N* = 96, traditional) and 2022 (*N* = 97, FC) cohorts (Fig. 2). Students’ perceptions of the flipped classroom model were positive. 88% of students reported that it was an effective approach to learning, while 80% of students in the flipped cohort agreed that more large-group sessions should incorporate course director-produced pre-work videos and most (74%) found the linked multiple-choice questions helpful. Accordingly, students’ perceptions of the value of pre-work were higher in the flipped classroom group than the traditional group (*U* = 3219.5, *p* < 0.001; *r* = 0.27); 86% of students in the flipped classroom agreed/strongly agreed that prework was useful compared to 58% in the traditional group.

## Discussion

Many studies have reported that the flipped classroom model is associated with a significant improvement in medical student test score performance. However, because cognitive domains are not typically assessed, it remains unclear if this reflects lower order recall or higher order mastery [4, 17, 18]. While the flipped classroom model encourages the development of higher-order skills, multiple choice exams may be insufficient to assess them [11, 14]. Because ZSOM has always used short-answer essay questions mapped to Bloom’s taxonomy in the pre-clerkship curriculum, our study was able measure the impact that traditional lecture and flipped classroom models had on the acquisition of facts as well as critical thinking in medical students. Our findings suggest that students in a flipped classroom learning environment gain foundational knowledge while improving higher order thinking.

It is noteworthy that some investigations fail to demonstrate significant flipped classroom driven improvement in student performance [19, 20]. In part, this has been attributed to poor student preparation and some conclude that this model may not justify its time investment for students and faculty [19]. This highlights an important point: flipped classroom effectiveness depends on its design and implementation. A well-structured flipped classroom delegates foundational cognitive domains of Bloom’s taxonomy to pre-class work, while class time is used for application, analysis, evaluation, and creation, with the opportunity for real-time feedback from instructors [8, 12, 14, 19].

Our findings are consistent with a growing body of literature showing that students generally report positive perceptions of the flipped classroom model [5, 11, 17]. Several studies have identified specific elements that students value including increased autonomy, opportunities for feedback, and meaningful engagement [10, 17]. If expectations are clearly communicated to students regarding the content and level of proficiency they must achieve prior to class, they are free to proceed at their own pace and in a manner that best complements their learning style [4, 14].

Several limitations should be considered when interpreting the results of our study. The retrospective, non-randomized design limits our findings to observations of differences between two cohorts without causal conclusions. Additionally, although each year’s examination assessed the same learning objectives, about 25% of the questions were changed from year to year, limiting direct comparison. This study was conducted within a single course at one institution, limiting generalizability, and our study is subject to selection bias. Lastly, our study did not include follow-up beyond the course period, limiting our ability to assess long-term impacts.

Although the flipped classroom model has been widely studied across health professions education, many of these studies include a mix of learners [18, 19, 21, 22], which makes it difficult to draw strong conclusions about its effectiveness in UME. More targeted research is needed to evaluate how the flipped classroom approach supports medical students. Furthermore, future studies must ensure that assessment strategies adequately measure higher-order cognitive outcomes, which aligns with the goal of the flipped classroom. Additionally, longitudinal studies would provide valuable insights into whether the observed benefits of this model translate into long-term competence.
